# Effects of Experiential Learning Programmes on Adolescent Prosocial Behaviour, Empathy, and Subjective Well-being: A Systematic Review and Meta-Analysis

**DOI:** 10.3389/fpsyg.2021.709699

**Published:** 2021-08-04

**Authors:** Hannah Hoi-Kiu Chan, Ho Yee Chloe Kwong, Geisty Lin Feng Shu, Chung Yan Ting, Frank Ho-Yin Lai

**Affiliations:** Department of Rehabilitation Sciences, The Hong Kong Polytechnic University, Hung Hom, Hong Kong

**Keywords:** experiential learning, prosocial behaviour, empathy, well-being, positive youth development, adolescence

## Abstract

**Introduction:** Effective adolescent learning programmes can positively influence adolescent development and curb risky behaviour. By immersing learners in an experience, experiential learning motivates learners to reflect on the experience to transform and create new skills, attitudes and ways of thinking. However, evidence of its effectiveness in learning programs facilitating positive youth development is still lacking. The objective of this study is to (a) identify the effect of adolescent learning programmes on prosocial behaviour, empathy and subjective well-being, (b) compare the effectiveness of experiential learning programmes and non-experiential learning programmes on improving these three outcomes, and (c) evaluating the effects of age on the outcomes of adolescent learning programmes.

**Methods:** This study was conducted following the Preferred Reporting Items for Systematic Reviews and Meta-Analyses (PRISMA) guidelines. Randomised controlled trials of learning programmes for typically developing adolescents aged 8–25 in the past 15 years were identified, and assessed for quality with the Physiotherapy Evidence Database (PEDRO) scale. One thousand ninety-six records were screened with the inclusion and exclusion criteria, and 20 studies were adopted for this meta-analysis. The standardised mean difference and 95% confidence interval (CI) of the effect of experiential learning program on empathy, prosocial behaviour, and subjective well-being were examined. Sub-group analysis based on age was conducted to examine the effects of experiential learning on adolescents in different stages of life.

**Results:** Experiential learning programmes were more effective than non-experiential learning programmes in improving empathy [d = 0.65 (0.07, 1.23)] and subjective well-being [d = 0.46 (0.33, 0.59)]. The effect sizes of the three outcomes in non-experiential learning programmes were non-significant. Studies conducted on older adolescents had the most significant improvements in the three outcomes.

**Conclusions:** Results suggest the broader application of experiential learning in adolescent learning programmes for older adolescents in the future to promote positive youth development.

## Introduction

Adolescence is a developmental period characterised by complex biological, psychological, social, emotional, sexual, and cognitive changes. This maturation offers an opportunity to foster a healthy and happy lifestyle as they transition to adulthood (Compas and Millstein, 1993). For example, their developing social cognition allows adolescents to grasp the complexities of social situations beyond simple rules, thus making room for new modes of prosocial behaviour with family members, peers, and the community (Fuligni, 2019). On the other hand, this period also leaves them vulnerable to developing problematic behaviours such as risky sexual behaviour, substance abuse, violence, addiction, and antisocial behaviours (Ciocanel et al., 2017). In recent years, though there is a drop in some adolescent risk behaviours, such as drug abuse, there is a worrying rise in internet and gaming addiction and risky sexual behaviour (Shek and Yu, 2011; Cheung and Cheung, 2019; Leung and Lin, 2019).

Adolescent prosociality, such as making small contributions to other members of society, promotes their personal social acceptance and integration for long-term functioning during adulthood (Fuligni, 2019). The transition of adolescents into thriving young adults raises educational and occupational prospects which enhances economic productivity (Fergusson et al., 2007). Addressing unique challenges during adolescence provides substantial personal benefits to health and well-being and also reduces economic costs for families and communities (Ciocanel et al., 2017). Positive youth development (PYD) is an ideology that aims to guide and empower youngsters by nurturing their self-efficacy, positive self-identity, psychosocial competence, and sense of belonging. Through providing adolescents with opportunities and resources to develop their strengths and form meaningful social networks, positive behaviour and the development of a prosocial and well-adjusted adult is promoted (Duncan et al., 2007). There are many examples of PYD programmes worldwide. Literature review concerning the outcomes of PYD programmes were done in the past with promising results (Catalano et al., 2004). Extensive research has been conducted by Shek and colleagues on the learning programme “Positive Adolescent Training through Holistic Social Programs” (*PATHS)* (Shek et al., 2010; Shek and Yu, 2011). Participants in *PATHS* displayed a lower level of substance abuse and delinquent behaviour than the control group (Shek and Yu, 2011) and also enabled development of positive interpersonal relationships, self-esteem, and sense of purpose (Shek et al., 2010). Other examples of PYD programmes include the Quantum Opportunities Program, Big Brother Big Sisters, Project K and the Summer Training and Education Program (Ciocanel et al., 2017).

In recent years, experiential learning (EXL) has been gaining popularity for its ability to engage students in active learning, translate classroom learning into real-world scenarios, and address community needs (Kruger et al., 2015). Currently, there is no single unanimous definition for the term “experiential learning” among researchers, but Kolb's Theory of Experiential Learning, which views learning as a process of creating knowledge through the transformation of experience (Kolb, 2014) is one of the most renowned theory supporting EXL due to his concrete theoretical base built upon the works of others. Theory of Experiential Learning states that for learning to occur, experience must first be grasped and then transformed through reflection and application. It involves four components that occur in a cyclical process: Concrete Experience (CE) is the opportunity for an experience, Reflective Observation (RO) makes sense of, breaks down, and transforms the experience through reflection, Abstract Conceptualisation (AC) is the formation of theoretical knowledge from which new behaviours and thinking emerge, and Active Experimentation (AE) is the practical application of new concepts (Kolb and Kolb, 2005; Alkan, 2016). This theory is built on the works of John Dewy, Kurt Lewin, Kurt Hahn, Jean Piaget, and so on (Dewey, 1986; James, 1990; Schein, 1996; Miettinen, 2000). While the works of Joplin (1981), Jarvis (2006) and Dean (1993) are alternative theories developed, Kolb's definition remains more inclusive of different modes of learning. For the purpose of this review, experiential learning programmes are thereby defined as learning programmes that include all four components of Kolb's learning cycle.

EXL is an effective form of learning as it engages the whole being through connecting the senses, intellect, and feelings during the learning process, which improves retention of information (Kolb and Kolb, 2005). Literature also points to EXL's effectiveness in improving the development of critical thinking (Lisko and O'dell, 2010) and personal insight (Burch et al., 2016). Through action-reflection and experience-abstraction, experiential learning is a process that facilitates learners to transform and create knowledge, skills, attitudes, and ways of thinking; thus, accommodate to various learning styles of individual learners by integrating the experience, perception, cognition, and behaviour (Lewis and Williams, 1994; Kolb and Kolb, 2009; Kolb, 2014). There is increasing inclusion of EXL concepts in learning programmes. For example, the Youth ImpACT Award is an example of an EXL programme that aims to promote PYD. This programme fosters participants' awareness of social issues through community engagement activities, whereby participants meet with community dwellers, explore the problems they face, and devise solutions using design thinking and innovative skills (Youth Impact Award, 2020).

Although literature asserts EXL's effectiveness in learning and skill development (Biers et al., 2006), there is a paucity of literature that specifically evaluates whether EXL programmes enhance PYD outcomes in adolescents. Moreover, what essentially constitutes an effective PYD programme is still obscure since there is no consensus about what components should be present in terms of programme design and targeted outcomes. For instance, the means of delivery, engagement methods, contexts, participants, and formats of the programmes (Brooks-Gunn and Roth, 2014; Tolan, 2016; Curran and Wexler, 2017).

Empathy, prosocial behaviour, and subjective well-being are key factors that influence healthy adolescent development and cultivate a harmonious society (Eisenberg and Miller, 1987; Silke et al., 2018). A wealth of literature suggests that empathy and prosocial behaviour play a key role in healthy social functioning and are particularly relevant in enhancing connectedness, cooperation, and understanding among people (Silke et al., 2018). For well-being, research shows that it is a reliable predictor of health and long-term positive adjustment (Gómez-López et al., 2019). As evidence suggests prosocial behaviour, empathy, and well-being are salient factors in facilitating PYD, they were chosen as the outcomes in this review. Prosocial behaviour is an umbrella term used to describe actions performed to enhance the welfare of others (Weinstein and Ryan, 2010; Spinrad and Eisenberg, 2017). It includes activities such as sharing, helping, caregiving, donating, volunteering, and acts of kindness. Empathy is the ability to apprehend others' emotional state (cognitive empathy) or the ability to share emotional experiences of others (affective empathy) (Eisenberg and Miller, 1987; Silke et al., 2018). Well-being is a multidimensional construct that includes psychological states such as positive affect or happiness, low negative affect or depression, and life-satisfaction (Curry et al., 2018). It also includes eudaimonic factors such as positive life functioning, self-actualisation, self-esteem, quality relationships, and purpose in life (Dodge et al., 2012; Moreira et al., 2015). Well-being will be referred to as “subjective well-being” in this review since all well-being outcomes were self-reported. The terminology “adolescent” traditionally refers to people under 19 years old (Curtis, 2015); however, recent literature suggests that the delayed transition to life stages such as education completion, career attainment, marriage, and parenthood expanded the length of adolescence into the mid-twenties (Mandarino, 2014; Sawyer et al., 2018). To increase the inclusiveness of this review, adolescents between the age of 8 to 25 will be explored.

The purpose of this systematic review and meta-analysis was to synthesise and categorise randomised controlled trials of adolescent learning programmes published between 2005 and 2020 into EXL programmes (those that incorporated the four elements of Kolb's Theory of Experiential Learning:) and non-experiential learning (NEXL) programmes (those that did not incorporate all four elements) and compare their effects on three PYD outcomes: prosocial behaviour, empathy, and subjective well-being. The time frame was selected as a comprehensive review on PYD programs was done on the years before (Catalano et al., 2004). The research objectives are (a) identifying the effect of adolescent EXL and NEXL programmes on prosocial behaviour, empathy and subjective well-being, (b) comparing the effectiveness of EXL and NEXL programmes on improving these three outcomes, and (c) evaluating the effects of age on the outcomes of adolescent learning programmes.

## Methods

### Guidelines

This study was conducted following the Preferred Reporting Items for Systematic Reviews and Meta-Analyses (PRISMA) guidelines (Page et al., 2020). This guideline has been widely accepted and endorsed in the academic community to facilitate accurate reporting in systematic reviews (Page and Moher, 2017). It serve as a standardised method to provide transparency to the full process behind the study, thus making this review replicable and hold it up to an internationally approved standard. The PRISMA 2020 checklist is included in the Supplementary Material.

### Identification of Studies

To identify suitable experimental studies, searches were conducted in 2020 on scientific databases: Wiley Online Library, ScienceDirect, PsycINFO, Taylor and Francis Online, SAGE Journals, and Springer LINK. A search was also conducted using Google Scholar. The search terms for interventions and outcomes were as follows.

**Figure 1:**
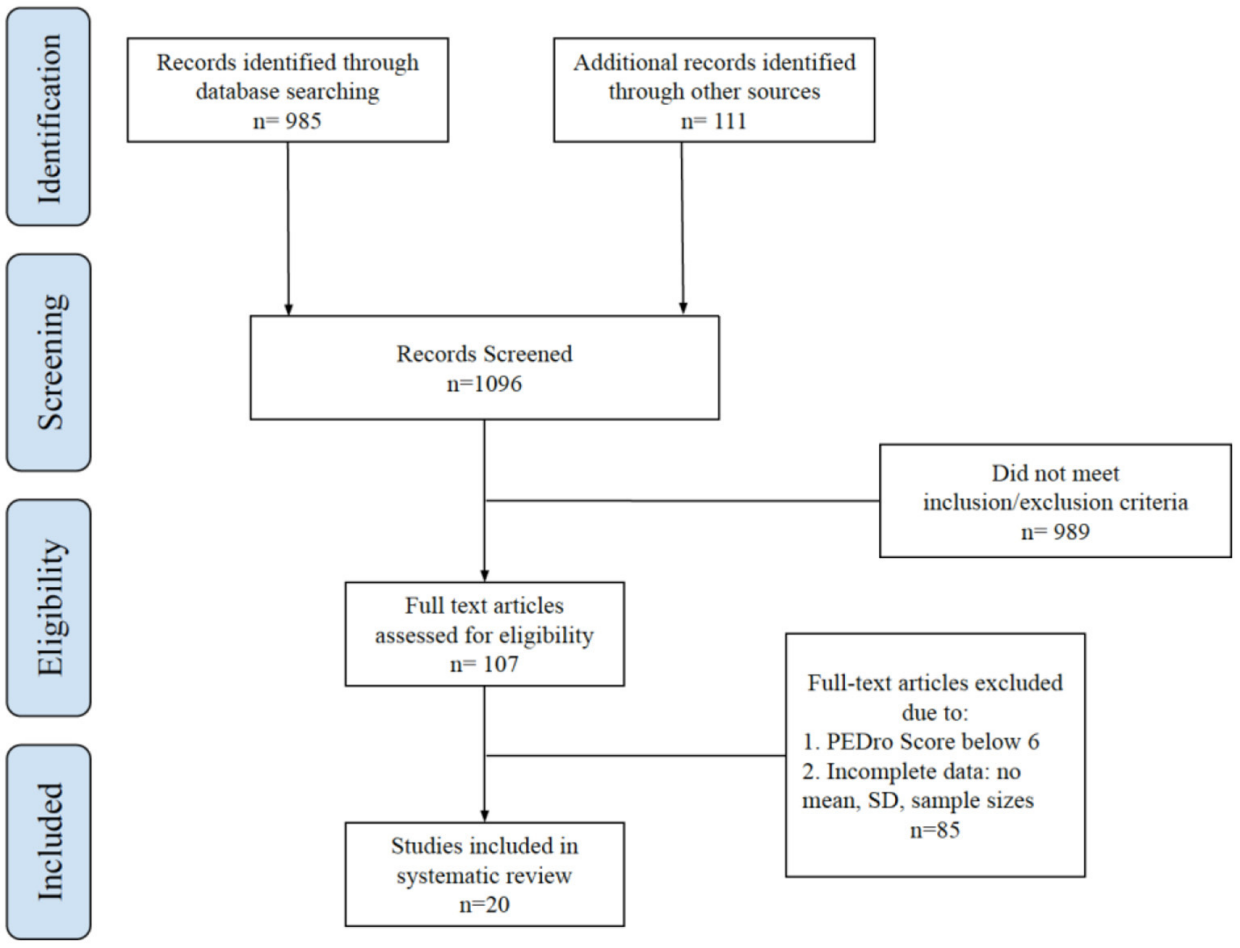
Flow chart illustrating the search and selection process of studies.

This search identified 985 articles, screened and categorised by four reviewers working independently unless a discussion is warranted. To this, we added 111 articles by following references in journal articles and checking systematic reviews. This initial set of 1,096 articles was screened by reading the titles and abstracts, and 906 articles were excluded. These articles had unrelated interventions or did not measure target outcomes. The remaining 190 potentially eligible articles were then assessed by full-text screening. Within, 83 articles were excluded for not meeting the inclusion criteria. The remaining 107 articles were read in full and rated according to the Physiotherapy Evidence Database (PEDro) Scale. Articles with a PEDro score of less than six were excluded. Articles that did not provide data such as mean, SD, and sample size, and convertable statistics to Cohen's d were also excluded (Bird, 2014; Nelson et al., 2015; Guo et al., 2018). By the end, 20 articles were left for the final systematic review. All four reviewers reviewed the full texts to ensure its eligibility. Please see Figure 1 for the flow diagram that summarises the literature search process.

### Inclusion and Exclusion Criteria

Studies were included in the review if they met the following criteria:

(a) Articles must focus on delivering learning programmes for adolescents between 8 and 25 years of age (or a mean sample age within this range).
(b) Articles must focus on intervention outcomes of empathy, prosocial behaviour, or subjective well-being.
(c) Articles must focus on the assessment of typically developing adolescents from non-clinical samples.
(d) Articles must report on randomised controlled trials, be peer-reviewed, written in the English language, and published in or after 2005.
(e) Articles failing to meet the above criteria (a–d) are included if effects for relevant comparison groups or sub-sample analyses were reported separately.

### Appraisal of Study Quality and Data Extraction

Potentially eligible articles were scored individually by four reviewers with the Physiotherapy Evidence Database (PEDro) Scale (Physiotherapy Evidence Database, 2020a). PEDro is designed to rate the validity of randomised controlled trials through critical appraisal (Moseley et al., 2019). The reviewers collaborated to assess and score the articles according to PEDro criteria. The items include (1) specified eligibility criteria, (2) randomisation of subjects, (3) concealed allocation, (4) similarity at baseline, blinding of subjects, (5) blinding of therapists, (6) blinding of assessors, (7) measures of at least one key outcome obtained from more than 85% of the subjects initially allocated to groups, (8) intention to treat, (9) results of between-group statistical comparisons reported for at least one key outcome, (10) and (11) point measures and measures of variability for at least one key outcome provided. Item 1 (specified eligibility criteria) is not used to calculate the PEDro score. For items 2–11, one point is given for each item when a criterion is clearly satisfied. The possible range of score is 0–10. Only papers with a score of six or above were included in the final systematic review as the Physiotherapy Evidence Database (2020b) states that scores between six and ten are moderate to high quality. All studies were also randomised controlled trials (RCT) because this type of experiment reduces bias and allows for a better examination of cause and effect due to having a control group and random subject allocation (National Institute for Health Care Excellence, 2020).

### Data Analysis

Standardised mean differences (Cohen's *d*) were computed from reported inferential or descriptive statistics using the Campbell Collaboration Standardised mean difference Calculator (Wilson, 2001) or extracted from articles when provided. Standardised mean differences were coded as positive values to suggest an increase in prosocial behaviour, empathy, or subjective well-being. Standardised mean differences were reverse coded for the well-being outcomes: depression, depressive symptoms and anxiety. The value of Cohen's *d* (later displayed as *d*) 0.20 indicates a small, 0.50 a medium, and 0.80 a large standardised mean difference (Cohen, 1988). When a study had multiple measures for the same outcome, an overall standardised mean difference was calculated by pooling the individual standardised mean differences. The pooled standardised mean differences were calculated with the calculator *Meta Essentials* (Suurmond et al., 2017). For studies with multiple time-points, we calculated the outcome closest to the time of intervention.

## Results

### Study Quality Assessment

The average PEDro score of articles in this review is 6.65 which reflects of moderate-to-high quality articles. The highest score of 8 was obtained in four studies, a score of 7 was obtained in five studies, and a score of 6 was obtained in eleven studies. Please refer to Table 1 for individual scores for each article.

**Table 1:** Main characteristics of studies included in the systematic review.

**Study**	**Intervention mode of delivery**	**Intervention programme**	**Control**	**Age (mean/range)**	**Location**	**Outcome**	**Outcome measurement tools**	**N1 (I)/N2(C)**	**PEDro score**
**Experiential learning (EXL)**									
Bosse et al. (2012)	University classroom (small training groups)	*Standardised/Simulated patient (SP)*: Interview with standardised patient, reflection and feedback, discuss, and debriefing	Seminar without additional training	24 (U)	Germany	E	Calgary-Cambridge Referenced Observation Guide [T]	33/34	6
						WB (self-efficacy)	(Self-created questionnaire) [S]		
Cooke et al. (2013)	University	*Goal setting intervention*: Talk on benefit and strategies to increase step count, reviewing step count and discussion, setting personal goals by writing them in a diary, and continuous recording	Same activity without review	22.2 (U)	UK	P (perceived behaviour control) P (intention to promote physical activity)	Questionnaire based on Theory of Planned Behaviour (TPB) [S] Motivational Interviewing Treatment Integrity (MITI) scoring tool [T]	70/66	8
Daeppen et al. (2012)	University (small groups)	*Motivational Interviewing (MI) Training* : Understand MI theory and mechanisms discussion, persuasion exercise, illustration (DVD), discussion/ didactics, structured ex, role plays, exercise (round robin)Recognise and reinforce change by Illustration (DVD), trainer demonstration, discussion role play, exercise (change talk jeopardy), team role plays	Training in basic communication skills	24.7 (U)	Switzerland	E	Balanced Emotional Empathy Scale (BEES) [S]	42/49	6
Deane et al. (2017)	School-based (12 people per group)	*Project K Youth Development Program*: 3-week wilderness adventure (team building and challenge-based activities), 10-day community service, workshops on topics related to youth health and well-being, and a 1-year adult mentoring	Adventure day	13–15 (S)	New Zealand	WB (academic self-efficacy) WB (social self-efficacy)	Self-Efficacy Questionnaire (SEQ) [S] Self-Efficacy Questionnaire (SEQ) [S]	482/417	6
Henry et al. (2011)	University (small groups)	*Ageing game*: instructions, briefing and transformation, the Ageing Game— Ageing Simulation period (5 stations), and debriefing of feelings and experience	Lecture with discussion	25 (U)	US	E	Empathy questions adapted from the Maxwell and Sullivan Survey [S]	62/62	7
Karasimopoulou et al. (2012)	Classroom/small groups	*Skills for Primary School Children*: Topics to develop students' personal and social skills. Each session includes introduction, 3 activities, evaluation, and homework.	Normal school curriculum	10–12 (P; grade 5–6)	Greece	WB (psychological) WB (Mood and feeling)	Psychological Well-being subscale of Kidscreen-52 Questionnaire [S] Mood and Feeling subscale of Kidscreen-52 Questionnaire [S]	128/158	6
						WB (self-perception)	Self-perception subscale of Kidscreen-52 Questionnaire [S]		
Lakin and Mahoney (2006)	School-based (small groups)	*Youth Community Service Program*: promote empowerment and sense of community. Includes skill building (6 sessions on social action, cooperation, leadership, and empathy), planning (6 sessions where participants chose a social problem they wish to address and plan action), action (community service), and regular discussion and reflection.	Normal school curriculum	10–13 (P; grade 6)	US	P (intent to be involved in future community action) P (Civic responsibility/sense of responsibility) E WB (global self-efficacy)	(Self-created questionnaire) [S] (Self-created scale) [S] Index of Empathy for Children and Adolescents [S] Cowen, Work, Hightower, Wyman, Parker, and Lotyczewski (1991) self-efficacy scale [S]	29/14	6
Li et al. (2013)	Community (6–8 children per small group)	Adventure-based Training Program: promote understanding towards the importance of psychosocial well-being and physical activities, stress and coping, and depression prevention. Includes 5 education sessions (health-related talks or workshops) and 1-day adventure-based training camp (Including warm up, briefing, group activities, team building, adventure-based games, and debriefing).	Leisure activities	11 (P; grade 5–6)	Hong Kong	WB (self-esteem) WB (quality of life) WB (anxiety) WB (depressive symptoms)	Rosenberg's Self-Esteem Scale (RSES) [S] Paediatric Quality of Life Inventory [S] Chinese Version of the State Anxiety Scale for Children (CSAS-C) [S] The Center for Epidemiologic Studies Depression Scale for Children (CES-DC) [S]	56/64	8
O'Hare et al. (2015)	After-school (15 children per group)	“*Mate-Tricks” Prosocial Behaviour After-School Program*: child, parent and family SEL sessions include snack time, opening game, review of previous session, and closing gam. The training provides theoretical framework, practical application, and sessions that include a combination of participation as well as reflection and sharing.	No treatment	9-10 (P)	Ireland	P (prosocial behaviour)	Peer Relations and Prosocial Behavior Questionnaire [S]	220/198	6
*Samuels et al. (2016)*	School-based (small groups)	*Humane Education Program: Circle of Compassion*: includes experiential activities and service-learning events, student centred activities, multimedia and discussion to explore challenges faced by pets, farm animals, wildlife, the environment. The children use what they learn to plan and implement strategies to help animals, other children, and the environment with continuous discussion.	Chess club	9–10 (P; grade 4)	US	P (prosocial behaviour)	Teacher Observation of Classroom Adaptation–Checklist (TOCA-C) [T]	119/48	6
**Non-experiential learning (NEXL)**									
Berger et al. (2018)	Classroom	*ERSAE-Stress-Prosocial (ESPS)*: SEL, stress-reduction and prosocial program that consists of warm-up, experimental work, psycho-educational knowledge, contemplative practise, learned skill and homework assignments (sharing and practise)	Social Studies class	12.46 (S)	Tanzania	P (prosocial behaviour) WB (anxiety)	Prosocial Subscale of Strengths and Difficulties Questionnaire (SDQ) [S] Spence Anxiety Scale for Children [S]	95/88	6
Connolly et al. (2018)	Classroom	*Roots of Empathy (ROE)*: SEL, mentalization program where children are instructed to (1) label the baby's feelings, (2) describe the baby's behaviour, (3) describe the links between the two, (4) label their own feelings towards the content or discontent baby, (5) describe how the mother cares for and helps the baby feel content	Normal school curriculum	8–9 (P; grade 4–7)	Ireland	P (prosocial behaviour) E WB (quality of life)	Strength and Difficulties Questionnaire (SDQ) rated by parents and teachers [T] Interpersonal Reactivity Index (IRI) [S] Child Health Utility-9D [S]	538/424	7
Ferri et al. (2019)	University (small groups)	*Expert Patient Teaching*: 2 theoretical seminars, 2 interactive meetings with nursing teacher and expert patient, and debriefing and reflection	Same activity without expert-patient involvement	20.9 (U)	Italy	E (Emotional Empathy) E (Perspective taking) E (compassionate care) E (standing in patient's shoes)	Balanced Emotional Empathy Scale (BEES) [S] Jefferson Scale of Empathy- Health Profession Student (JSE-HPS) [S] Jefferson Scale of Empathy—Health Profession Student (JSE-HPS) [S] Jefferson Scale of Empathy—Health Profession Student (JSE-HPS) [S]	72/72	8
Herrera et al. (2011)	Community (one-on-one)	*Big Brothers Big Sisters School-based Mentoring*: creative activities (e.g., drawing, arts, and crafts), games, discussions, and academic activity	No treatment	11.23 (P)	US	WB (global self-worth)	Global Self-Worth subscale of the Self-Esteem Questionnaire [S]	565/574	7
Horowitz et al. (2007)	Classroom (small groups)	*Interpersonal Psychotherapy–Adolescent Skills Training program (IPT–AST)*: Psychoeducation, CB program that educates about the nature and risk for depression and teaches how to (a) monitor daily moods; (b) identify activating events; (c) discover, challenge, realistically evaluate, and revise negative beliefs; (d) recognise the connexions among activating events, beliefs, and consequences (e.g., affect and behaviours); and (e) problem solve and cope with stressful events. Participant workbook is provided for homework.	Normal school curriculum	14.43 (S; grade 7–10)	US	WB (depressive symptoms) WB (depressive symptoms)	Children's Depression Inventory (CDI) [S] The Center for Epidemiological Studies Depression Scale (CES-D) [S]	112/169	6
Humphrey et al. (2016)	Classroom	*Promoting Alternative Thinking Strategies (PATHS)*: SEL program with taught activities that aims to help students manage their behaviour, understand their emotions, and work well with others. Each class contains lessons and send-home activities that cover topics such as identifying and labelling feelings, controlling impulses, reducing stress and understanding other people's perspectives, in addition to associated physical resources and artefacts (e.g., posters, feelings, dictionaries).	Normal school curriculum	7–9 (P; year 3–5)	UK	P (prosocial behaviour) P (cooperation) P (responsibility) E	Prosocial Subscale of Strengths and Difficulties Questionnaire (SDQ) [T] Social Skills Improvement System subscales (SSIS) [S] Social Skills Improvement System subscales (SSIS) [S] Social Skills Improvement System subscales (SSIS) [S]	2,340/2,176	7
Kolić-Vehovec et al. (2020)	School-based (individual)	*School of Empathy*: game including various social situations in school in which the players had to choose the reactions they find the most suitable. The player will shift from the victim role to the bystander role, then bully role once they finish the tasks at each stage.	Another game related to safe use of internet	12-14 (S)	Spain, Malta, UK, Ireland	P (appropriate assertive reaction) P (assertiveness)	(Game metrics with frequencies of correct reactions) [T] Children's Assertive Behaviour Scale (CABS) [S]	77/61	6
Morton and Montgomery (2012)	Community (group-based)	*Questscope non-formal education (QS NFE):* non-formal education program aimed to empower adolescents. It consists of educational (dialogue-based learning) and social (recreational, cultural, and vocational activities) sessions, and reflection in a prosocial environment.	Waitlist	13–15 (S)	Jordan	P WB (self-efficacy) WB (emotional symptoms)	Prosocial Subscale of Strengths and Difficulties Questionnaire (SDQ) [S] General Self-Efficacy (GSE) Scale [S] Emotional Symptoms subscale of Strengths and Difficulties Questionnaire (SDQ)[S]	67/60	7
Schonert-Reichl et al. (2015)	Classroom	*MindUP*: SEL program that consists of 12 lessons on mindfulness, self-regulation and caring for others. It also includes lessons that involve performing acts of kindness for one another and collectively engaging in community service-learning activities	Normal school curriculum (social responsibility program)	10.24 (P; grade 4–5)	Canada	P (social responsibility) E E (perspective taking) WB (optimism) WB (emotional control) WB (depressive symptoms)	Social Goals Questionnaire [S] Interpersonal Reactivity Index [S] Interpersonal Reactivity Index [S] Optimism subscale of Resiliency Inventory [S] Emotional Control subscale of Resiliency Inventory [S] Depressive Symptoms subscale of Seattle Personality Questionnaire for Children [S]	48/51	8
						WB (school self-concept)	Marsh's Self-Description Questionnaire [S]		
Stallard et al. (2014)	Classroom	*FRIENDS*: CB, Anxiety Prevention Program that consists of teacher teaching the children how to reduce anxiety using the acronym “FRIENDS.”	Normal school curriculum	9-10 (P; year 4–5)	UK	WB (anxiety and depressive symptoms) WB (worry) WB (self-esteem) WB (life satisfaction)	Revised Child Anxiety and Depression Scale [S] Penn State Worry Questionnaire for Children [S] Rosenberg Self-Esteem Scale [S] Subjective Well-being Assessment [S]	449/372	6

^*^ Standardised mean differences had been coded as positive values which suggests an increase in prosocial behaviour (P), empathy (E), or subjective well-being (WB).

*Age description: P = Primary school-age (8–12 years old), S = Secondary school-age (12–18 years old), U = University-age (18–25 years old).*

*Outcome measure types: S = Self-reported, T = Third party measures (e.g., third-party observations, teacher-rated, parents-rated)*.

### Study Characteristics

#### Design

Presented in Table 2 are the characteristics of the 20 studies included in this review. There were ten studies on EXL and ten studies on NEXL interventions. The sample size ranged from 43 to 4,516. All ten of the EXL programmes were carried out in developed countries, such as the United States, Canada, the United Kingdom, and Greece. Eight NEXL programmes were carried out in developed western countries such as the United States, Canada, and the United Kingdom, while the remaining two were conducted in Tanzania and Jordan. Seven studies compared the intervention group with a control group using a standard school curriculum, twelve used an alternative intervention, and three used no intervention or wait-list as the control. Please see Appendix A for more details of the studies.

**Table 2:** Search terms included in the systematic review.

**Category**	**Search terms**
Experiential learning	Experiential learning OR action learning OR education programme OR education program OR learning programme OR learning program OR school programme OR school program
Prosocial behaviour	Prosocial behaviour OR prosocial behaviour OR prosocial acts OR prosocial responding OR prosocial intention OR helping intention OR helping behaviour OR helping behaviour OR kindness
Empathy	Empathy OR compassion
Subjective Well-being	Subjective well-being OR well-being OR positive affect OR happiness OR life satisfaction
Randomised controlled trial	Randomised controlled trial OR randomised controlled trial OR RCT OR control

#### Participants

The total number of randomised participants was 10,761. There were 2,351 participants in EXL programmes (intervention *n* = 1,241; control *n* = 1,110) and 8,410 participants in NEXL programmes (intervention *n* = 4,363; control *n* = 4,047). The subjects were categorised into three age groups: primary school-age (ages 8–12), secondary school-age (ages 12–18), and university-age (ages 18–25). The EXL sample consisted of five studies with primary school-age subjects, one with secondary school-age subjects, and four with university-age subjects. The NEXL sample consisted of five studies with primary school-age subjects, four with secondary school-age subjects, and one with university-age subjects. The average proportion of male subjects included is 44.58% in EXL programmes and 49.17% in NEXL programmes. Participants from eighteen studies were recruited from educational institutions, while participants from two studies were recruited from the community.

### Intervention

Programmes were categorised into EXL and NEXL programmes by reviewers based on the criteria as described in the introduction. Five of the ten EXL programmes reported they were based on the Theory of Experiential Learning, while the other five were categorised by reviewers. Both types of programmes varied in programme duration, structure, and number and demographic of participants. The activities in the EXL programmes vary widely. For example, they are based on community service (Deane et al., 2017), simulation of physical disabilities (Henry et al., 2011) and classroom games and discussion sessions about understanding one's emotions and social skills (O'Hare et al., 2015). The activities in NEXL programmes are more similar in nature, as most are classroom games and discussions (Horowitz et al., 2007; Humphrey et al., 2016; Connolly et al., 2018). Please see Appendix A for more information on the learning programmes.

### Outcome Measures

Outcome measures related to prosocial behaviour, empathy, and subjective well-being were selected to be included in the systematic review. A majority of studies used self-reported measures while some used third-party measures, such as the Teacher Observation of Classroom Adaptation–Checklist (TOCA-C) (Samuels et al., 2016) and the Strengths and Difficulties Questionnaire (SDQ) (Prosocial Behaviour Subscale) (Humphrey et al., 2016).

Self-reported measures and third-party measures were used to measure prosocial behaviour. The Prosocial Subscale of Strengths and Difficulties Questionnaire (SDQ) was used in four studies. Self-reported and third-party measures were used to measure empathy. The Interpersonal Reactivity Index (IRI) was used in two studies, while all other measures were different in each study. All measures on subjective well-being were self-reported. There were three measures for quality of life and life satisfaction, six measures for self-esteem and self-efficacy, and ten measures for mood and affect related items. Rosenberg's Self-Esteem Scale (RSES) and the Center for Epidemiological Studies Depression Scale (CES-D) were each used in two studies. In total, 14 articles used self-reported measures only, 2 used third-party measures only, and 4 used both self-reported and third-party measures.

### Intervention Effects

**Figure 2:**
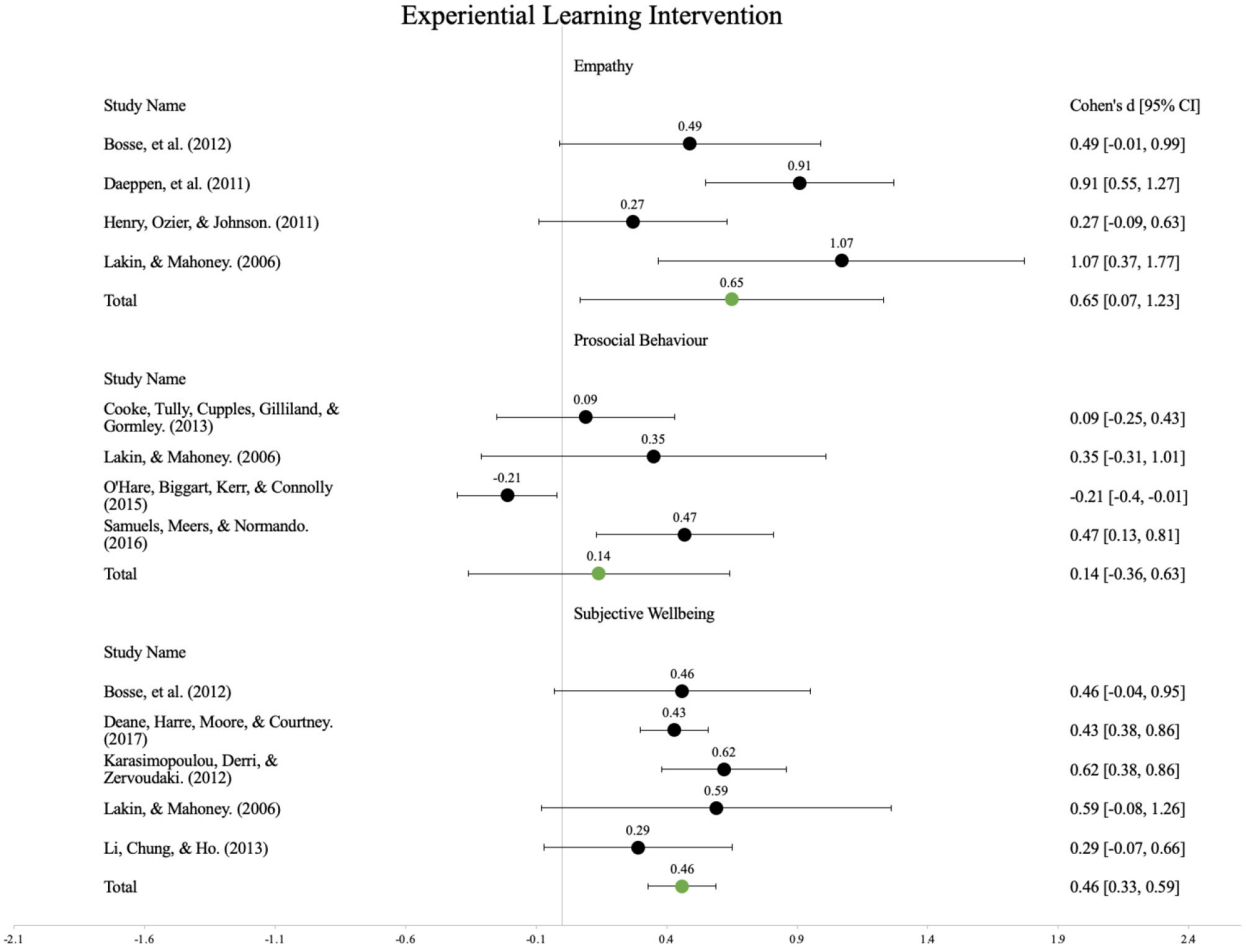
Forest plot showing the standardised mean difference of experiential learning interventions.

**Figure 3:**
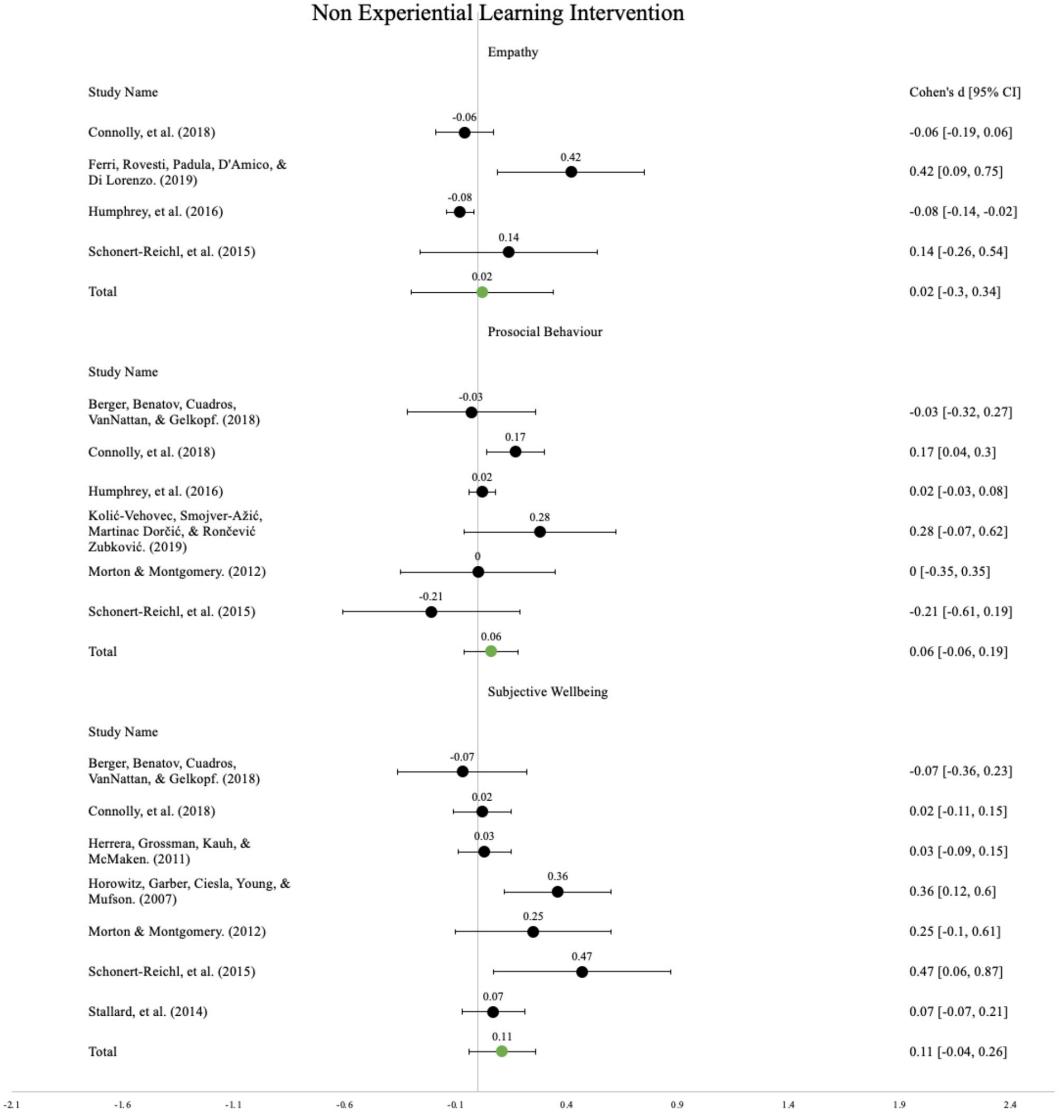
Forest plot showing the standardised mean difference of non-experiential learning interventions.

Six separate main effects analyses were conducted for EXL and NEXL programmes with respect to the three outcome categories. Please refer to Figures 2, 3 for the forest plots.

#### Experiential Learning Programmes

##### Empathy

Based on the empathy outcome measures reported in four studies, the overall standardised mean difference was *d* = 0.65, 95% CI (0.07, 1.23). This represents a moderately large effect concerning the observed increase in empathy outcomes associated with the participation in EXL programmes.

##### Prosocial Behaviour

Based on the prosocial behaviour outcome measures reported in four studies, the overall standardised mean difference was *d* = 0.14, 95% CI (−0.36, 0.63). This represents a non-significant effect concerning the observed increase in prosocial behaviour outcomes associated with the participation in EXL programmes.

##### Subjective Well-being

Based on the subjective well-being outcome measures reported in five studies, the overall standardised mean difference was *d* = 0.46, 95% CI (0.33, 0.59). This represents a moderate effect concerning the observed increase in subjective well-being outcomes associated with the participation in EXL programmes.

#### Non-experiential Learning Programmes

##### Empathy

Based on the empathy outcome measures reported in four studies, the overall standardised mean difference was *d* = 0.02, 95% CI (−0.3, 0.34). This represents non-significant effect concerning the empathy outcomes associated with the participation in NEXL programmes.

##### Prosocial Behaviour

Based on the prosocial behaviour outcome measures reported in six studies, the overall standardised mean difference was *d* = 0.06, 95% CI (−0.06, 0.19). This represents a non-significant effect concerning the observed increase in prosocial behaviour outcomes associated with the participation in NEXL programmes.

##### Subjective Well-being

Based on the subjective well-being outcome measures reported in seven studies, the overall standardised mean difference was *d* = 0.11, 95% CI (−0.04, 0.26). This represents a non-significant effect concerning the observed increase in subjective well-being outcomes associated with the participation in NEXL programmes.

#### Sub-group Analysis on Age

Main effects analyses were conducted for primary school-age, secondary school-age, and university-age participants with respect to the three outcome categories. Results indicate that all effects are non-significant, except for that of participants in the university-age range for empathy which *d* = 0.52, 95% CI (0.07, 0.97). Please refer to Table 3 for the results.

**Table 3:** Effect of age on intervention outcomes.

	**Primary school-age (8–12 years old)**	**Secondary school-age (12–18 years old)**	**University-age (18–25 years old)**
Empathy	0.03 (−0.48, 0.55) (*n* = 4)	–	0.52 (0.07, 0.97) (*n* = 4)
Prosocial behaviour	0.06 (−0.20, 0.32) (*n* = 6)	0.07 (−0.33, 0.48)(*n* = 3)	0.09 (−0.25, 0.43) (*n* = 1)
Subjective well-being	0.23 (−0.01, 0.47) (*n* = 7)	0.27 (−0.09, 0.62)(*n* = 4)	0.46 (−0.04, 0.95) (*n* = 1)

*n, number of studies*.

#### Sub-group Analysis on Control

Main effects analyses were conducted for controlled conditions with respect to the three outcome categories. Please refer to Table 4 for the results.

**Table 4:** Effects of control conditions on outcomes.

	**Alternative treatment**	**No treatment/waitlist**
Empathy	0.31 (−0.03, 0.65) (*n* = 8)	–
Prosocial behaviour	0.11 (0.04, 0.26) (*n* = 8)	−0.16 (−1.27, 0.96) (*n* = 2)
Subjective well-being	0.29 (0.12, 0.46) (*n* = 10)	0.08 (−1.06, 1.21) (*n* = 2)

*n = number of studies*.

## Discussion

### Effectiveness of EXL and NEXL Programmes

Results demonstrated that EXL programmes were effective in improving adolescents' empathy, prosocial behaviour, and subjective well-being. The following section suggests possible reasons for this based on information provided by the studies and literature on EXL.

Kolb's Theory of Experiential Learning is constructed based on the works of leading psychologists Kurt Lewin, William James, Jean Piaget, Lev Vygotsky, and others who shaped the field of human development and learning (Kolb, 2014). Components in the Theory of Experiential Learning are also explicitly structured to aid the transformation of experience into new ways of thinking and behaviour. This model of learning is likely a decisive factor for programme effectiveness because the four components of learning (Concrete Experience, Reflective Observation, Abstract Conceptualisation, Active Experimentation) helped participants assimilate and comprehend new knowledge. On the other hand, NEXL programmes do not fit Kolb's framework. Still, most NEXL programmes are constructed based on other well-established theories, such as Social-Emotional Learning and Cognitive-Behavioural Theory.

One major difference noted between the two types of learning programme was the amount of facilitation in reflection. With regards to the Reflective Observation component of Kolb's Theory of Experiential Learning, all EXL programmes had teachers, instructors, mentors or group members facilitating participants' reflection of new experiences while all NEXL programmes did not. EXL programmes that had a facilitator guide members reflect on their experiences systematically produced the most significant outcomes in empathy (Lakin and Mahoney, 2006; Daeppen et al., 2012). Many EXL programmes conducted Reflective Observation through verbal discussions of feelings, thoughts, and experiences, such as what participants learnt and how they think or felt they performed in an activity (Lakin and Mahoney, 2006; Bosse et al., 2012; Daeppen et al., 2012; Cooke et al., 2013; Li et al., 2013; O'Hare et al., 2015; Samuels et al., 2016). Some programmes crafted opportunities for self-reflection during the activity (Deane et al., 2017), two programmes used written methods (Lakin and Mahoney, 2006; Karasimopoulou et al., 2012), and one programme used open-ended questions to guide participants' reflection (Henry et al., 2011).

In contrast, instructors in NEXL programmes did not guide their participants to reflect. Two factors, namely the mode of education and the large participant size, may have contributed to the lack of reflection. In half of the NEXL programmes, didactic lectures or classroom-based teaching was the prime mode of education (Horowitz et al., 2007; Stallard et al., 2014; Schonert-Reichl et al., 2015; Humphrey et al., 2016; Berger et al., 2018), so the participants adopted a relatively passive mode of learning. On the other hand, half of the NEXL interventions were conducted as a class, while more than half of the EXL programmes were conducted in small groups. Whole classroom interventions may pose difficulties for teachers to guide students' reflection because of limited time and resources. Overall, results suggest that reflection is essential in improving programme outcomes.

In terms of Active Experimentation, the setting in which experimentation and application of new concepts was conducted differed between the two types of programmes. Participants in EXL programmes engaged with groups and individuals in the community (Lakin and Mahoney, 2006; Henry et al., 2011; Bosse et al., 2012; Daeppen et al., 2012; Karasimopoulou et al., 2012; Deane et al., 2017). Results show that EXL programmes that used service-learning events, community service projects, simulation, role-play, or adventure programmes to apply new concepts and skills were the most effective in increasing empathy, prosocial behaviour and subjective well-being outcomes.

In contrast, most NEXL programmes either lacked the Active Experimentation component, or if they did, limited participants' experimentation to the classroom without engaging the real-world (Horowitz et al., 2007; Stallard et al., 2014; Humphrey et al., 2016; Berger et al., 2018). This may limit effective learning as it prevents newly learnt concepts from being reconstructed in a meaningful and authentic context for deeper reflections (Jeyaraj, 2019). Ultimately, participants in most NEXL programmes had no significant improvements in prosocial behaviour and empathy. Notably, two NEXL programmes that provided community-immersive activities demonstrated significant improvements in empathy (Ferri et al., 2019), prosocial behaviour (Connolly et al., 2018) and subjective well-being outcomes (Schonert-Reichl et al., 2015). Overall, these results suggest that the opportunity for participants to apply concepts beyond the classroom may yield better programme outcomes. This is consistent with existing research as interacting with others facilitates the development of empathetic attitudes and emotional sensitivity (Carlo et al., 2015; Spinrad and Eisenberg, 2017).

The flexibility of programmes for participants to take ownership of activities differed significantly between NEXL and EXL programmes. Most EXL programmes were flexible, and teachers imposed few restrictions. Emphasis was placed on the learner's self-motivation to initiate, plan and implement a course of action to achieve goals (Lakin and Mahoney, 2006; Bosse et al., 2012; Daeppen et al., 2012; Karasimopoulou et al., 2012; Li et al., 2013,?; Samuels et al., 2016; Deane et al., 2017). Participants of these programmes had marked improvements in empathy, prosocial behaviour, and subjective well-being.

The NEXL programmes were often highly structured and teachers were required to follow programme guidelines to ensure programme fidelity (Horowitz et al., 2007; Stallard et al., 2014; Schonert-Reichl et al., 2015; Humphrey et al., 2016; Berger et al., 2018; Connolly et al., 2018). The authoritarian role of teachers and overreliance on traditional didactic teaching methods may have halted participants' self-discovery and engagement, leading to poor outcomes in NEXL programmes. Notably, an NEXL intervention that enabled participants to negotiate activities with their mentors had significant intervention effects on participants' subjective well-being (Morton and Montgomery, 2012). Hence, flexible programmes that provide opportunities for participants to take control of their learning may yield better outcomes.

It is worth noting that despite the relative ineffectiveness of NEXL programmes in improving empathy, prosocial behaviour and subjective well-being, it cannot be overlooked that out of the three outcomes, subjective well-being improved the most, albeit an less than small standardised mean difference (*d* = 0.11). One possible explanation is that most NEXL programmes aimed to teach adolescents how to regulate and understand their emotions, manage their behaviours, and reduce anxiety or depression (Horowitz et al., 2007; Stallard et al., 2014; Schonert-Reichl et al., 2015; Humphrey et al., 2016; Berger et al., 2018). These programmes were more self-oriented, focusing on understanding one's feelings and behaviours rather than helping others. Thus, improvement in subjective well-being was greater than empathy and prosocial behaviour in NEXL programmes.

### Effects of Age

Results show that university-age subjects benefitted most from the learning programmes, with a higher overall standardised mean difference than that of other age groups in all three outcomes (empathy, *d* = 0.52; prosocial behaviour, *d* = 0.09; and well-being, *d* = 0.46).

One reason university-age adolescents had the most profound improvements after attending learning programmes may be that they are more cognitively developed. Major growth spurts in the brain during puberty cause the emergence of new neuronal pathways, pruning of existing neural networks, and significant development of the prefrontal cortex. Based on *Piaget's Theory of Cognitive Development*, these neuronal changes enable teens to perform formal operational skills such as abstract thinking and hypothetico-deductive reasoning, thus improving their ability to think critically about abstract concepts such as morality and free will (Weiten, 2013; Boyd and Bee, 2015). Moreover, increasingly challenging cognitive tasks demanded by higher education further develop metacognitive skills, facilitating conscious control of thought and reflective thinking (Boyd and Bee, 2015).

In contrast, primary school-age adolescents are closer to the concrete operational stage. In this stage, children can think logically, but can only apply logic to tangible objects and events (Weiten, 2013). They have difficulty thinking hypothetically and understanding things they cannot see or have not experienced (Boyd and Bee, 2015). The development of empathy and prosocial behaviour is highly linked to age. As children age and their theory of mind develops, their ability to self-regulate and differentiate themselves from others improves. This increases their ability to empathise with others and respond prosocially (Carlo et al., 2015). The results of this review are consistent with existing research as empathy and prosocial outcomes were the greatest for university-age, moderate for secondary school-age, and lowest for primary school-age participants.

### Limitations and Implications

There are several limitations associated with this review. Firstly, as the interest of this review was to collect RCTs to ensure intervention reliability, the number of articles synthesised was limited. Only ten articles each were collated for NEXL and EXL, which may not be adequate to draw reliable conclusions on the overall effectiveness of these programmes. Secondly, the use of the PEDro scale to evaluate studies for internal validity may be too stringent. Since it is difficult to blind the administers, assessors and participants of the programs, many potential studies were screened out as a result of their low PEDro score. Thirdly, many factors contribute to the effectiveness of learning programmes beyond EXL or NEXL such as its activities, method of implementation, quality, and duration. Previous research also attests that participant characteristics such as age, gender, personality, experiences, and relationship with parents and peers also influence their empathetic responding, prosocial behaviour, and well-being (Lai et al., 2015; Silke et al., 2018). Hence, it would be inapt to attribute changes in outcomes to NEXL or EXL alone. Fourthly, this review looked at the immediate effects of learning programmes on the three outcomes, it is not clear what the long-term effects of these interventions may be.

The results of this review suggest that EXL programmes have a higher potential to improve adolescent empathy, prosocial behaviour and well-being than NEXL programmes. This encourages the broader application of EXL in learning programmes to enhance Positive Youth Development outcomes. Future learning programmes may incorporate components such as facilitated individual or small-group reflections, active experimentation in various settings, and flexibility on the learning programme along with promotion of self-motivation to potentially increase the effectiveness of the programmes to promote PYD outcomes. However, since most EXL interventions in this review were conducted on older adolescents, further RCTs of EXL programmes for adolescents between ages 12-18 is recommended to confirm the effects of EXL programmes. Furthermore, only three studies measured all three outcomes. More RCTs of learning programmes that measure empathy, prosocial behaviour and subjective well-being is recommended.

## Conclusion

Adolescence is a critical time for implementing learning programmes that promote PYD. Moreover, the use of such learning programmes may reduce risky behaviours in youths that lead to adverse physical and psychosocial effects that carry on into adulthood. The results of this review support the use of EXL programmes in schools and communities to develop empathy and subjective well-being. More RCTs on EXL programmes for adolescents are needed to deepen our understanding of how they can help adolescents thrive.

## Data Availability Statement

The raw data supporting the conclusions of this article will be made available by the authors, without undue reservation.

## Author Contributions

FL is responsible for supervising the process of paper writing. All authors contributed to this article and approved the submitted version.

## Conflict of Interest

The authors declare that the research was conducted in the absence of any commercial or financial relationships that could be construed as a potential conflict of interest.

## Publisher's Note

All claims expressed in this article are solely those of the authors and do not necessarily represent those of their affiliated organizations, or those of the publisher, the editors and the reviewers. Any product that may be evaluated in this article, or claim that may be made by its manufacturer, is not guaranteed or endorsed by the publisher.
