# Supplementary material for: Effects of Experiential Learning Programmes on Adolescent Prosocial Behaviour, Empathy, and Subjective Well-being: A Systematic Review and Meta-Analysis
Source: Front Psychol. 2021 Aug 4;12:709699. doi: 10.3389/fpsyg.2021.709699 (PMC8371439; doi:10.3389/fpsyg.2021.709699)
Supplement: Supplementary file 2 [file Data_Sheet_2.docx]

Appendix A

Details of Studies Included in the Systematic Review

| Study | Program Name | Interven-tion | Control | Age(mean/ range) | Location | Setting (Format) | Program Activities | Outcome | N1 (I) | N2 (C) | d | Duration/ Frequency | % of males | Pedro score |
| --- | --- | --- | --- | --- | --- | --- | --- | --- | --- | --- | --- | --- | --- | --- |
| Berger et al. (2018) | ERSAE-Stress-Prosocial (ESPS) | NEXL | Social Studies class | 12.46 (S) | Tanzania | Classroom | An SEL, stress-reduction & prosocial program that consists of warm-up, experimental work, psycho-educational knowledge, contemplative practice, learned skill and homework assignments (sharing & practice) | P (prosocial behavior) | 95 | 88 | -0.0257 | 2 weeks (45 min per week) | 49.2 | 6 |
|  |  |  |  |  |  |  |  | WB (anxiety) |  |  | 0.0652* |  |  |  |
| Bosse et al. (2012) | Standardized/ Simulated patient (SP) | EXL | Seminar without additional training | 24 (U) | Germany | University (small training groups: 1 tutor, 3 students) | Multiple sessions of interview with standardized patient, reflection & feedback, discuss, and debriefing | E | 33 | 34 | 0.4856 | 3 weeks | 54.4 | 6 |
|  |  |  |  |  |  |  |  | WB (self-efficacy) |  |  | 0.4565 |  |  |  |
| Connolly et al. (2018) | Roots of Empathy (ROE) | NEXL | Normal school curriculum | 8-9 (P; grade 4-7) | Ireland | Classroom | An SEL, mentalization program where children are instructed to 1) label the baby's feelings, 2) describe the baby's behavior, 3) describe the links between the two, 4) label their own feelings towards the content or discontent baby, 5) describe how the mother cares for and helps the baby feel content | P (prosocial behavior) | 538 | 424 | 0.1682 | 9 months (once per month, 27 lessons) | 51.4 | 7 |
|  |  |  |  |  |  |  |  | E |  |  | -0.064 |  |  |  |
|  |  |  |  |  |  |  |  | WB (quality of life) |  |  | 0.017 |  |  |  |
| Cooke et al. (2013) | n/a (Goal setting intervention) | EXL | Same activity without review | 22.2 (U) | UK | University | A program that consists of a talk on benefit & strategies to increase step count, reviewing step count and discussion, setting personal goals by writing them in a diary, and continuous recording | P (perceived behavior control) | 70 | 66 | 0.1479 | 4 weeks | 40.4 | 8 |
|  |  |  |  |  |  |  |  | P (intention to promote physical activity) |  |  | 0.023 |  |  |  |
| Daeppenet al. (2011) | Motivational Interviewing (MI) Training | EXL | Training in basic communication skills | 24.7 (U) | Switzerland | University (small groups) | A program that consists of two sessions: Session 1) Understand MI theory & Mechanisms Discussion, persuasion exercise, illustration (DVD), discussion/ didactics, structured ex, role plays, exercise (round robin)  Session 2) Recognize & reinforce change by Illustration (DVD), trainer demonstration, discussion role play, exercise (change talk jeopardy), team role plays | E | 42 | 49 | 0.9149 | 2 sessions (4 hours each) | 40.7 | 6 |
| Deane, et al. (2017) | Project K Youth Development Program | EXL | Adventure day | 13-15 (S) | New Zealand | School-based  (12 people per group) | A program that consists of a 3-week wilderness adventure (team building & challenge-based activities), 10-day community service (PB), workshops on topics related to youth health & wellbeing, and a 1-year mentoring (adult mentor, set 4 goals) | WB (academic self- efficacy) | 482 | 417 | 0.4273 | 14 months (3 weeks of wilderness adventure, 10 days of community service component, and 1 year of mentoring) | 53.75 | 6 |
|  |  |  |  |  |  |  |  | WB (social self- efficacy) |  |  | 0.4416 |  |  |  |
| Ferri et al. (2019) | Expert Patient Teaching | NEXL | Same activity without expert-patient involvement | 20.9 (U) | Italy | University (small groups) | Consisted of 2 theoretical seminars, 2 interactive meetings with nursing teacher &  expert patient, and debriefing & reflection | E (Emotional Empathy) | 72 | 72 | 0.4371 | 6 months (two 1.5hours theoretical seminar, two 2hours interactive meeting, 1hour debriefing) | 18.1 | 8 |
|  |  |  |  |  |  |  |  | E (Perspective taking) |  |  | 0.6714 |  |  |  |
|  |  |  |  |  |  |  |  | E (compassionate care) |  |  | 0.6188 |  |  |  |
|  |  |  |  |  |  |  |  | E (standing in patient’s shoes) |  |  | -0.057 |  |  |  |
| Henry et al. (2011) | Aging Game | EXL | Lecture with discussion | 25 (U) | US | University (small groups) | An Aging game that consists of instructions, briefing & transformation, the Aging Game–– Aging Simulation period (5 stations), and debriefing of feelings and experience | E | 62 | 62 | 0.2662 | 90minutes (15-30mins intro, 45-60mins Aging Game, 15-30mins debrief) | 16 | 7 |
| Herrera et al. (2011) | Big Brothers Big Sisters School-based Mentoring | NEXL | No treatment | 11.23 (P) | US | Community (One-on-one) | A one-on-one mentoring program that consists of creative activities (e.g. drawing, arts & crafts), games, discussions, and academic activity | WB (global self-worth) | 565 | 574 | 0.03 | 4.9 months average (meeting an average of 3.1 times per month) | 45 | 7 |
| Horowitz et al. (2007) | Interpersonal Psychotherapy–Adolescent Skills Training program (IPT–AST) | NEXL | Normal school curriculum | 14.43 (S; grade 7-10) | US | Classroom (small groups) | A Psychoeducation, CB program that educates about the nature and risk for depression and teaches how to (a) monitor daily moods; (b) identify activating events; (c) discover, challenge, realistically evaluate, and revise negative beliefs; (d) recognize the connections among activating events, beliefs, and consequences (e.g., affect and behaviors); and(e) problem solve and cope with stressful events. Participant workbook is provided for homework. | WB (depressive symptoms) | 112 | 169 | -0.4138* | 8 weeks (90-min weekly sessions) | 45 | 6 |
|  |  |  |  |  |  |  |  | WB (depressive symptoms) |  |  | -0.3075* |  |  |  |
| Humphrey et al. (2016) | Promoting Alternative Thinking Strategies (PATHS) | NEXL | Normal school curriculum | 7-9 (P; year 3-5) | UK | Classroom | An SEL program with taught activities that aims to help students manage their behavior, understand their emotions, and work well with others.  Each class contains lessons and send-home activities that cover topics such as identifying and labeling feelings, controlling impulses, reducing stress and understanding other people's perspectives, in addition to associated physical resources and artifacts (e.g., posters, feelings, dictionaries). | P (prosocial behavior) | 2340 | 2176 | 0.0985 | 2 years | 51.2 | 7 |
|  |  |  |  |  |  |  |  | P (cooperation) |  |  | 0.0083 |  |  |  |
|  |  |  |  |  |  |  |  | P (responsibility) |  |  | -0.0326 |  |  |  |
|  |  |  |  |  |  |  |  | E |  |  | -0.0798 |  |  |  |
| Karasimopoulou et al. (2012) | Skills for Primary School Children | EXL | Normal school curriculum | 10-12 (P; grade 5-6) | Greece | Classroom or small groups | A program that consists of sessions with different topics that aims to develop students' personal and social skills.  Each session includes introduction, 3 activities, evaluation, and homework (choose a positive action and a person that needs it (PB)) | WB (psychological) | 128 | 158 | 0.1075 | 23 weeks (one 45 min lesson per week) | 48.6 | 6 |
|  |  |  |  |  |  |  |  | WB (Mood & feeling) |  |  | 1.5855 |  |  |  |
|  |  |  |  |  |  |  |  | WB (self-perception) |  |  | 0.1797 |  |  |  |
| Kolić‐Vehovec et al. (2019) | School of Empathy | NEXL | Another game related to safe use of internet | 12-14 (S) | Spain, Malta, UK, Ireland | School-based (individual) | The game consisted of various social situations in school in which the players had to choose the reactions they find the most suitable. The player will shift from the victim role to the bystander role, then bully role once they finish the tasks at each stage. | P (appropriate assertive reaction) | 77 | 61 | 0.2658 | 6 weeks (total of 6 hours) | 52.2 | 6 |
|  |  |  |  |  |  |  |  | P(assertiveness) |  |  | 0.2849 |  |  |  |
| Lakin, & Mahoney(2006) | Youth Community Service Program | EXL | Normal school curriculum | 10-13 (P; grade 6) | US | School-based (small groups) | A youth community service program that aims at promoting empowerment and sense of community. It consists of skill building (6 sessions on social action, cooperation,  leadership, and empathy), planning (6 sessions where participants chose a social problem they wish to address and plan action), action (community service, PB), and regular discussion & reflection (verbal or written). | P (intent to be involved in future community action) | 29 | 14 | 0.6959 | 10 weeks (20 sessions) | 35.2 | 6 |
|  |  |  |  |  |  |  |  | P (Civic responsibility/ sense of responsibility) |  |  | 0 |  |  |  |
|  |  |  |  |  |  |  |  | E |  |  | 1.0666 |  |  |  |
|  |  |  |  |  |  |  |  | WB (global self-efficacy) |  |  | 0.5909 |  |  |  |
| Li et al. (2013) | Adventure-based Training Program | EXL | Leisure activities | 11 (P; grade 5-6) | Hong Kong | Community (small groups with 6-8 children) | The adventure training program aims to help participants understand the importance of psychosocial wellbeing and physical activities, stress and coping, and depression prevention. It consisted of 5 education sessions (health-related talks or workshops) and 1-day adventure-based training camp (Including warm up, briefing, group activities, team building, adventure-based games, and debriefing). | WB (self-esteem) | 56 | 64 | 0.3292 | 1 academic year (5 education sessions) | 52.5 | 8 |
|  |  |  |  |  |  |  |  | WB (quality of life) |  |  | 0.1132 |  |  |  |
|  |  |  |  |  |  |  |  | WB (anxiety) |  |  | -0.247* |  |  |  |
|  |  |  |  |  |  |  |  | WB (depressive symptoms) |  |  | -0.4749* |  |  |  |
| Morton & Montgomery (2012) | Questscope non-formal education (QS NFE) | NEXL | Waitlist | 9-10 (P) | Jordan | Community (group-based) | A non-formal education program aimed to empower adolescents. It consists of educational (dialogue-based learning) and social (recreational, cultural, and vocational activities) sessions, and reflection in a prosocial environment. | P | 67 | 60 | 0 | 2 years (three 8-month education cycles, 5 days per week) | 85 | 7 |
|  |  |  |  |  |  |  |  | WB (self-efficacy) |  |  | 0.2161 |  |  |  |
|  |  |  |  |  |  |  |  | WB (emotional symptoms) |  |  | 0.2926 |  |  |  |
| O'Hare et al., (2015) | “Mate-Tricks” Prosocial Behavior After-School Program | EXL | No treatment | 9-10 (P) | Ireland | After-school (15 children per group) | An SEL after-school program sessions that consists of child, parent and family sessions. It includes snack time, opening game, review of previous session, and closing gam. The training provides theoretical framework, practical application, and sessions that include a combination of participation as well as reflection and sharing. | P (prosocial behavior) | 220 | 198 | -0.2055 | 1 academic year (1 1/2 hours per session that contains several 10–20-minute activities) | 55.3 | 6 |
| Samuels et al. (2016) | Humane Education Program: Circle of Compassion | EXL | Chess club | 9-10 (P; grade 4) | US | School-based (small groups) | The program used experiential activities and service-learning events, student centered activities, multimedia and discussion to explore challenges faced by pets, farm animals, wildlife, the environment. The children use what they learn to plan and implement strategies to help animals, other children, and the environment with continuous discussion. | P (prosocial behavior) | 119 | 48 | 0.4674 | 11 weeks (once per week) | 48.9 | 6 |
| Schonert-Reichl et al. (2015) | MindUP | NEXL | Normal school curriculum  (social responsibility program) | 10.24 (P; grade 4-5) | Canada | Classroom | An SEL program that consists of 12 lessons on mindfulness, self-regulation and caring for others. It also includes lessons that involve performing acts of kindness for one another and collectively engaging in community service-learning activities (PB). | P (social responsibility) | 48 | 51 | -0.2106 | 12 weeks (approximately once per week, 40 –50 min per class) | 46 | 8 |
|  |  |  |  |  |  |  |  | E |  |  | 0.08 |  |  |  |
|  |  |  |  |  |  |  |  | E (perspective taking) |  |  | 0.194 |  |  |  |
|  |  |  |  |  |  |  |  | WB (optimism) |  |  | 0.4487 |  |  |  |
|  |  |  |  |  |  |  |  | WB (emotional control) |  |  | 0.6095 |  |  |  |
|  |  |  |  |  |  |  |  | WB (depressive symptoms) |  |  | -0.3436* |  |  |  |
|  |  |  |  |  |  |  |  | WB (school self-concept) |  |  | 0.463 |  |  |  |
| Stallard et al. (2014) | FRIENDS | NEXL | Normal school curriculum | 9-10 (P; year 4-5) | UK | Classroom | A CB, Anxiety Prevention Program that consists of teacher teaching the children how to reduce anxiety using the acronym "FRIENDS."  F Feelings  R Remember to relax  I I can do it. I can try my best  E Explore solutions and coping step plans  N Now reward yourself. You’ve done your best  D Don’t forget practice  S Smile. Stay calm for life | WB (anxiety & depressive symptoms) | 449 | 372 | -0.1962* | 9 weeks (one 60 min session per week) | 48.6 | 6 |
|  |  |  |  |  |  |  |  | WB (worry) |  |  | -0.1024* |  |  |  |
|  |  |  |  |  |  |  |  | WB (self-esteem) |  |  | 0.0049 |  |  |  |
|  |  |  |  |  |  |  |  | WB (life satisfaction) |  |  | -0.0134 |  |  |  |

*Table A1. Details of studies included in the systematic review.*

*Note.* P (Primary school age or mean age 8-12), S (Secondary school age or mean age= 12-18), U (University age or mean age 18-25); SEL (Social Emotional Learning), CB (Cognitive Behavioral), PB (prosocial behavior)

* Effect sizes had been coded as positive values in the systematic review which suggests an increase in prosocial behavior (P), empathy (E), or subjective wellbeing (WB)
